# Modification on acute myocardial infarction model through left anterior descending coronary artery ligation: An experimental study on rats (*Rattus norvegicus*) using readily available materials

**DOI:** 10.14202/vetworld.2019.1448-1453

**Published:** 2019-09

**Authors:** Johanes Nugroho, Wiwik Misaco Yuniarti, Ardyan Wardhana, Cornelia Ghea

**Affiliations:** 1 Department of Cardiology and Vascular Medicine, Faculty of Medicine, Universitas Airlangga, Surabaya, Indonesia; 2 Department of Cardiology and Vascular Medicine, Dr. Soetomo General Hospital, Surabaya, Indonesia; 3 Department of Veterinary Clinical Science, Faculty of Veterinary, Universitas Airlangga, Surabaya, Indonesia; 4 PILAR Research and Education, Cambridge, UK

**Keywords:** left anterior descending coronary artery ligation, myocardial infarction, rat model

## Abstract

**Background and Aim:**

Several difficulties are involved in creating models for myocardial infarction (MI) in animals, such as low survival rates after acute MI, complicated techniques in creating animal models, complexities in confirming acute MI incidence, and complex surgical tools needed in the process. This study aimed to develop an animal model for acute MI using Wistar rats utilizing simple instruments that are readily available in standard animal laboratories.

**Materials and Methods:**

We induced MI in 48 Wistar rats using the left anterior descending coronary artery ligation modification technique without tracheal incision and ventilator. This ligation technique was performed 1-2 mm distal to the left atrial appendage. MI occurrence was evaluated using heart enzyme parameters 24 h post-ligation and histological studies of the infarcted area 6 weeks after the ligation. Rats were divided into the coronary artery ligation group and sham group.

**Results:**

Of the 48 rats, 24 (50%) died within 24 h post-ligation, but no further deaths occurred in the next follow-up period of 6 weeks. The average infarct size in six rats within 24 h of ligation was 35%±5.7%. The serum glutamic oxaloacetic transaminase level of the group treated with coronary artery ligation was statistically significantly higher than that of the sham group (p=0.000).

**Conclusion:**

We developed an MI rat model with consistent infarction size, in which the long-term death of rats was not observed. Our ligation technique for an MI rat model can be a reference for experimental settings without ventilators for small animals.

## Introduction

Animal models of acute myocardial infarction (MI) are very important for studying cellular and molecular changes in the heart. However, various difficulties are associated with creating animal MI models, such as low survival rates after acute MI, complicated techniques in creating animal models, complexities in confirming acute MI incidence, and complex surgical tools needed in the process.

Albino rats (*Rattus norvegicus*) are frequently used for animal models because their care is easy and cost-effective [1]. A commonly utilized modeling method is left anterior descending (LAD) coronary artery ligation. However, this method has its own challenges, such as high mortality rate (~27%) and large variance in the size of the infarction (15-38%) [2]. One of the challenges of using MI rat models is the intubation technique, as rats have relatively small airways.

This study aimed to develop an acute MI animal model using Wistar rats, without making a tracheal incision for intubation, and using simple instruments that are readily available in standard animal laboratories.

## Materials and Methods

### Ethical approval

This study was conducted at the Model Animal Laboratory of the Department of Biochemistry and the Electron Microscope Laboratory of the Faculty of Medicine. The care and usage of animals conformed with the principles of laboratory animal care, and adequate measures were taken to minimize pain. The protocol was approved by the Ethical Committee of the Faculty of Veterinary Medicine (approval no. 184-KE).

### Induction of MI

The study included 48 14-week-old male Wistar rats, weighing an average of 200-300 g. All rats were randomly distributed into the following three groups: Group I - LAD coronary artery ligation and sacrificed 1 day post-ligation; Group II - LAD coronary artery ligation, with a 2-week recovery period followed by a 4-week observational period, and sacrificed thereafter; Group III - LAD coronary artery ligation, with a 2-week recovery period, swimming practice 5 times a week for 4 weeks, with individually set practice duration (40% of the maximum duration), and sacrificed thereafter; and Group IV (sham group) - no LAD coronary artery ligation treatment, but underwent thoracotomy, and sacrificed after 6 weeks. All rats were kept in plastic cages (three rats in each cage), with a 12:12-h dark/light cycle and average temperature of 22°C-27°C. Pellet feed and water were supplied *ad libitum*.

**Figure-1:**
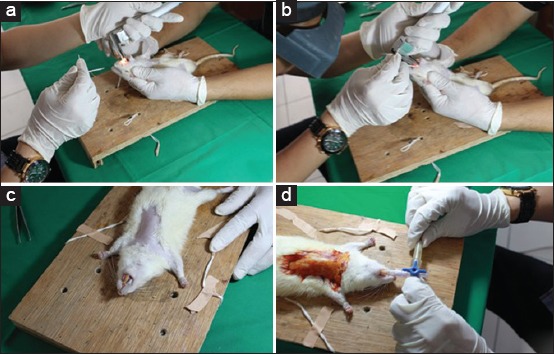
Intubation process: (a) Direct laryngoscopy was performed using a neonatal laryngoscope. A wire was inserted inside a 14-gauge intravenous catheter. (b) Insertion of the intravenous catheter with the help of an assistant to place the head into the “floating” position. (c) The wire was then detached from the intravenous catheter after ensuring that the intravenous catheter had entered the lumen of the trachea. (d) Once the intravenous catheter was inside the lumen of the trachea, the intravenous catheter was connected to a three-way stopcock and an oxygen cannula attached to an oxygen tank.

Prior to LAD coronary artery ligation, the rats were intramuscularly anesthetized with a combination of ketamine hydrochloride (50 mg/kg body weight) and xylazine (5 mg/kg body weight). Skin color, respiratory pattern, and level of consciousness were monitored during the anesthetic procedure. The rats were then intubated using 14-gauge intravenous catheters with small wire stylets. Direct laryngoscopy was performed using a small (for neonates) laryngoscope (Figure-1). The stylet was then detached from the intravenous catheter after ensuring that the intravenous catheter had entered the lumen of the trachea. Once the intravenous catheter was inside the lumen of the trachea, contact was made with a jagged structure, and this was further confirmed with the expansion of the chest when positive oxygen was administered. The intravenous catheter was then connected to a three-way stopcock. One port was connected to an oxygen cannula attached to an oxygen tank. Oxygen was supplied at a rate of 0.5 L/min. To maintain lung pressure and prevent lung collapse during thoracotomy, the open/close procedure was applied to the third port with a frequency of 70-80 times/min to attain a tidal volume of 1.5-3 mL.

Electrocardiogram (ECG) measurement, using an ECG machine (Cardisuny C110; Fukuda M-E Kogyo Co., Ltd., Chiba, Japan) with modified electrodes, was performed prior to the thoracotomy. Leads were attached to the four extremities, with one to the precordial anterolateral area of the rats. ECG was recorded in leads I, II, III, aVR, aVL, aVF, and V6, at a speed of 50 mm/s. ECG measurement was repeated 24 h post-ligation. Results from both measurements were then compared.

**Figure-2:**
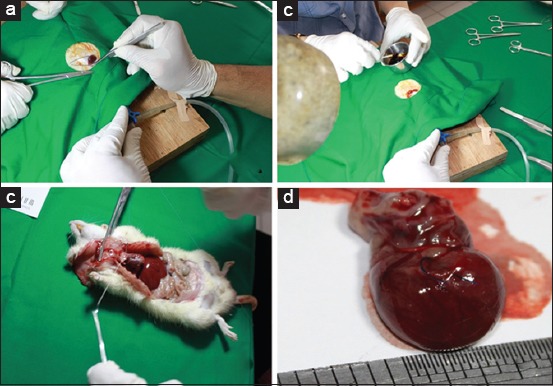
Coronary artery ligation and extraction of the heart: (a) exposing the heart during coronary artery ligation procedure; (b) closing the surgical area after artery ligation; (c) heart extraction for sacrifice procedure; (d) the extracted heart showing the ligation 1-2 mm distal of the left atrial appendage.

Thoracotomy was performed by making a small incision using small surgical scissors while separating the skin and muscle tissues on the left parasternal of the 4^th^ and 5^th^ intercostal spaces (Figure-2). Then, a speculum was attached to maintain fixation of the 4^th^ and 5^th^ intercostal spaces. Once the heart and lungs were visible, the pericardium was then separated carefully. The heart was then pushed from a laterocaudal to a cranial position, keeping it slightly out of the thoracic cavity so that the LAD coronary artery could be easily identified.

Once the LAD coronary artery was identified, ligation was performed using Prolene^®^ 6/0 thread (Ethicon, Somerville, NJ, USA), 1-2 mm distal of the left atrial appendage. An LAD coronary artery ligation was confirmed successfully if a pale color change was observed in the distal area of the heart, on which the anterolateral ligation was made. The ribs were then stitched back together using a Prolene 2/0 thread, whereas the skin was stitched using a Prolene 6/0 thread. Subsequently, the rat was extubated, once adequate spontaneous ventilation had been observed. The incision wound was treated with sterile gauze dressing and daily application of povidone-iodine solution. Stitches were removed after 7-10 days.

### Post-MI evaluation

Acute MI was diagnosed by a change in the ST segment or Q wave on lead V6, I, or aVL in the ECG 1 day post-ligation, compared with the ECG prior to ligation. Creatine kinase isoenzyme muscle–brain (CKMB) serum and serum glutamic oxaloacetic transaminase (SGOT) levels were measured 1 day post-ligation. Survival rate was calculated based on the percentage of surviving rats 6 weeks post-ligation.

Six weeks post-ligation, the rats were sacrificed in an anesthetized state using ketamine hydrochloride (50 mg/kg body weight). The chest cavity was opened from the middle of the chest, and the heart was excised by tying the aorta ascendens. The excised heart was placed in phosphate-buffered saline (10%) solution. Thereafter, morphological examination was performed macroscopically, and specimens for histopathological examination were prepared.

The infarcted region was stained with triphenyl tetrazolium chloride (TTC) for the macroscopic examination of the infarct size [3]. The heart was transversally incised into three parts, each having a thickness of 10 mm, starting from the distal part of the ligation area. All parts were cleaned with saline, stained with TTC, and incubated for 20 min at 37°C. Viable hearts appeared maroon, and the infarcted region appeared paler or whitish.

**Figure-3:**
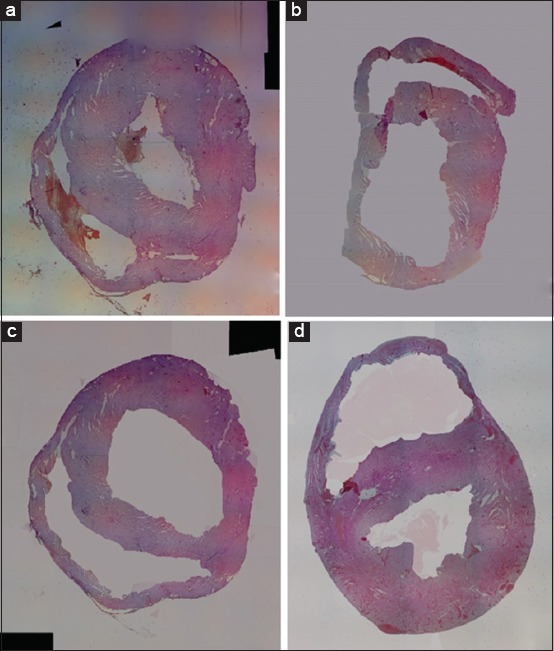
Myocardial infarction size measurement of groups (a) I, (b) II, (c) III, and (d) IV (hematoxylin and eosin stain, 40× magnification).

**Figure-4:**
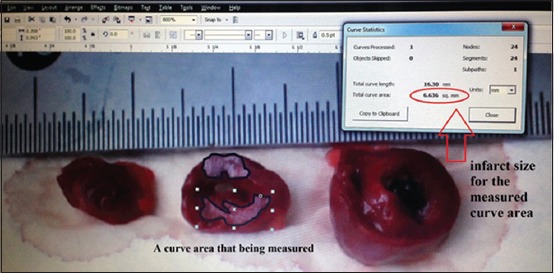
Macroscopic measurement of the infarcted area using graphic software.

The MI area was measured in the hematoxylin and eosin-stained histopathological specimens (Figure-3). Each section was photographed, and the width of the infarcted area was marked and calculated using a graphic software (CorelDRAW^®^ Graphics Suite X5; Corel Corporation, Ottawa, ON, Canada) (Figure-4). Infarct size was expressed as the percentage of the infarcted area against the total transversal left ventricle area.

### Statistical analysis

Data were expressed as mean ± standard deviation. Differences within groups were analyzed with one-way analysis of variance. In addition, differences among groups were analyzed with Mann–Whitney U-test. The test results were considered statistically significant if p<0.05.

## Results

Twenty-four rats (50%) died within 24 h following the procedure. However, none of the remaining 24 rats died during the course of the following 42 days until the time they were sacrificed. Table-1 shows no significant difference in body weight among the groups (p=0.14). The SGOT of the groups (Groups I-III) treated with LAD coronary artery ligation was statistically significantly higher than that of the sham group (Group IV) (p<0.01) (Table-2). Meanwhile, analysis of the CKMB level showed no statistically significant difference between the two groups (p=0.09).

**Table 1:** Mean weight of the subjects.

Group	n	Weight (g)	p-value
I	6	237.5±25.05	0.60
II	6	231.67±16.33	
III	6	242.50±19.43	
IV	6	245.67±10.89	

Values are presented as mean±standard deviation

**Table 2:** SGOT and CKMB levels.

Group	n	SGOT		CKMB	
	
		Mean±SD	p-value	Mean±SD	p-value
Ligation	18	435.22±168.56	<0.01	2476.54±957.21	0.09
Sham	6	162.84±39.12		1589.53±729.91	

CKMB=Creatine kinase isoenzyme muscle–brain, SD=Standard deviation, SGOT=Serum glutamic oxaloacetic transaminase

Results of the microscopic examination of the infarct area showed that Group II had the largest average infarcted area (56.5%), whereas Group I had the smallest infarcted area (35.0%). The mean infarct areas of Groups III and IV were 44.3% and 0.0%, respectively. Macroscopic inspection of the specimens prepared with TTC showed infarct areas of 33% (Group I), 54% (Group II), 45% (Group III), and 0% (Group IV), thus confirming the microscopic findings.

The infarct size in Groups I-III, which underwent LAD coronary artery ligation, ranged from 28% to 59%. Table-3 shows statistically significant differences in infarct size within the groups (p<0.01). The increase in infarct size in Group III compared with that of Group I was 21.5%. Meanwhile, Group III showed a 9.3% increase in infarction size. Differential testing showed that the mean infarct area in Group III was statistically significantly lower than that of Group II (without exercise) (p<0.01).

**Table 3:** Infarcted area of the ligation groups.

Group	Infarct size (%)	p-value
I	35.0±5.7	<0.01^a^
II	56.5±2.5	<0.01^b^
III	44.3±2.9	
IV	0±0	

Values are presented as mean±standard deviation.

a Differences among Groups I, II, and III,

b Differences between Groups II and III

## Discussion

We developed an MI rat model with successful MI induction on all rats with a survival rate of 50% within 24 h post-ligation. The mortality rate of this model was still higher than that of other standard models (5-40% mortality rates) [4]. Deaths during the 1^st^ day post-MI are mostly associated with ventricular tachycardia/fibrillation conditions post-ligation [5], while acute heart failure and rupture are the common causes of postoperative death during days 3-7 post-MI [6,7].

This MI model was developed with modified methods in response to the unavailability of instruments such as laryngoscope and ventilator that are suitable for Wistar rats. Furthermore, to our knowledge, the procedures implemented in the development of this model have never been done before. Such procedures included intubation using neonatal laryngoscope and intravenous cannula and manual ventilation using a three-way stopcock connected to an oxygen tank. No tracheal incision was required in our procedure. Therefore, we were able to avoid the risks of tracheal edema and bleeding related to tracheostomy [8]. Another modification was intubation using transcutaneous tracheal illumination without laryngoscope [9]. Although the procedure is less traumatic, it requires another operator to retract the tongue and perform transillumination to provide better visualization. Another simple method of intubation using the oropharyngeal intubation wedge from a common 3-mL syringe was proposed by Jou *et al*. [10].

Although the heart was less accessible and less exposed in our thoracotomy technique with parasternal incision, there was less bleeding [11]. Another alternative incision via the midsternum would enable more access to the heart, but would cause more bleeding [12]. The quality of thoracotomy to induce coronary occlusion directly influences study outcomes. Minimally invasive thoracotomy through the intercostal space is recommended for an MI model to reduce the confounding effects of the surgical procedure on inflammation [13]. This study showed an increase in the SGOT levels of the ligation groups. However, no significant differences were observed in the CKMB levels between the ligation and sham groups. Chest muscle damage and heart injuries caused by the thoracotomy techniques presumably triggered the increase in CKMB levels without heart ischemia [14]. Srikanth *et al*. [15] reported that the CKMB level in the sham group was similar to that in the healthy group, but it was significantly lower than that in the MI group. Minimizing injury to the heart by avoiding excoriation and heart immobilization using an earbud might contribute to a normal CKMB level in the sham group.

There were some challenges associated with this model. Hypersalivation during laryngoscopy obscured the laryngeal view. Antisialogogue administration would be beneficial. If laryngospasm occurred, we recommend allowing the animal to recover for 15 min. In our experience, laryngospasm happened after the second unsuccessful intubation. After intubation, the intravenous catheter should be secured safely, as it can easily dislodge because there was no cuff. Finally, the part of the LAD to be ligated must be definitely not too distal. Ligating the LAD at the same anatomical location across groups is important. However, as the LAD was not easily identified, we used left atrial appendage as guidance.

In this study, the infarct size measured 1 day post-ligation was 35%. This size was reported not to cause any malfunction of the left ventricle, which explains the absence of congestive heart failure-related death within 42 days post-ligation [16]. Moreover, LAD coronary artery ligation in this study was performed more distally (i.e., 1-2 mm below the left atrial appendage) to minimize variation in the infarcted area [17]. LAD coronary artery ligation in the proximal area was reported to have 4-65% variance in the infarcted area [18]. Moreover, artery ligation performed very close to the origin was reported to have a 24-h mortality rate of 100% and produced >65% larger infarct size [19]. Expansion of the infarcted area occurs over time. The infarct area of MI rats with no exercise (Group II) 6 weeks post-ligation was 56.5%. In addition to time, variance in infarct size may be caused by the measurement method in relation to certain areas because of the resorption and retraction of the infarcted area and hypertrophy of the non-infarcted region [20].

Despite the merits of our LAD ligation technique, some limitations should be noted. First, we did not perform imaging studies such as echocardiography or gadolinium-enhanced magnetic resonance imaging to select rats with consistent infarct sizes [21,22]. It is important to emphasize that infarct size does not differ between groups before exercise intervention. Second, we did not evaluate if the deaths within the first 24 h post-MI were due to surgical errors or very large infarct sizes. Third, we did not perform specific pharmacological intervention that would validate this model. Finally, we did not perform ECG assessment, apply oxygen saturation and end-tidal carbon dioxide, and control the temperature during the anesthetic procedure. Any undetected hypercarbia and hypoxia during the procedure would influence the outcomes.

## Conclusion

We have developed an MI rat model with consistent infarction size, and long-term death of rats was not observed. Our LAD ligation technique to develop an MI rat model can be a reference for experimental settings without ventilators for small animals. When applied in human clinical practice, this model may be useful for evaluating the mechanism of the expansion of the left ventricle MI area after an acute MI, especially in patients who are not receiving reperfusion and are performing light exercise.

## Authors’ Contributions

JN conceived, conducted the research work, performed the investigations, and reviewed the manuscript. WMY designed and supervised the study and reviewed the manuscript. AW analyzed the data, performed statistical analysis, and drafted the manuscript. CG conducted literature search and data acquisition. All authors read and approved the final manuscript.
